# Supplementary material for: Interdependence of cellular and network properties in respiratory rhythmogenesis
Source: bioRxiv. 2023 Nov 2:2023.10.30.564834. Preprint. [Version 1] doi: 10.1101/2023.10.30.564834 (PMC10634953; doi:10.1101/2023.10.30.564834)
Supplement: 1 [file NIHPP2023.10.30.564834v1-supplement-1.pdf]

bioRxiv preprint doi: https://doi.org/10.1101/2023.10.30.564834; this version posted November 2, 2023. The copyright holder for this preprint (which was not certified by peer review) is the author/funder, who has granted bioRxiv a license to display the preprint in perpetuity. It is made available under aCC-BY 4.0 International license.

970 # SUPPLEMENTARY MATERIAL

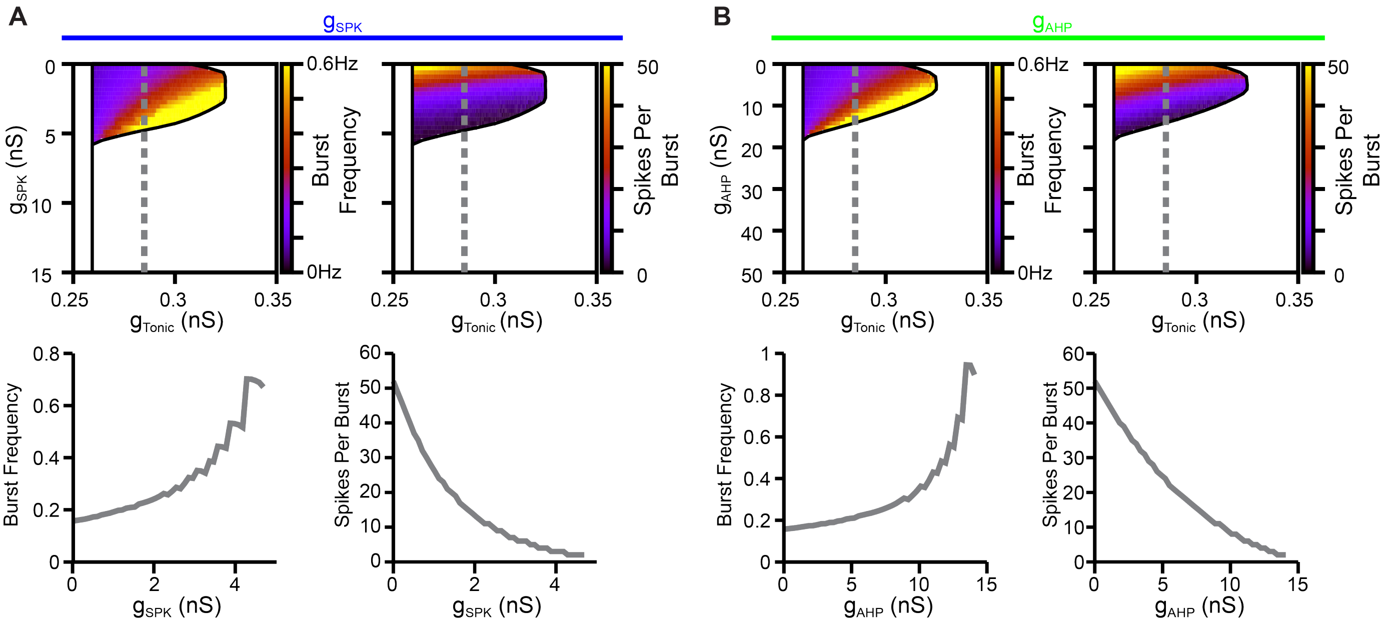

Figure 1 Supplement 1. Effect of changes in (A) g_{SPK} or (B) g_{AHP} on burst frequency (left) and the number of spikes per burst (right).

bioRxiv preprint doi: https://doi.org/10.1101/2023.10.30.564834; this version posted November 2, 2023. The copyright holder for this preprint (which was not certified by peer review) is the author/funder, who has granted bioRxiv a license to display the preprint in perpetuity. It is made available under aCC-BY 4.0 International license.

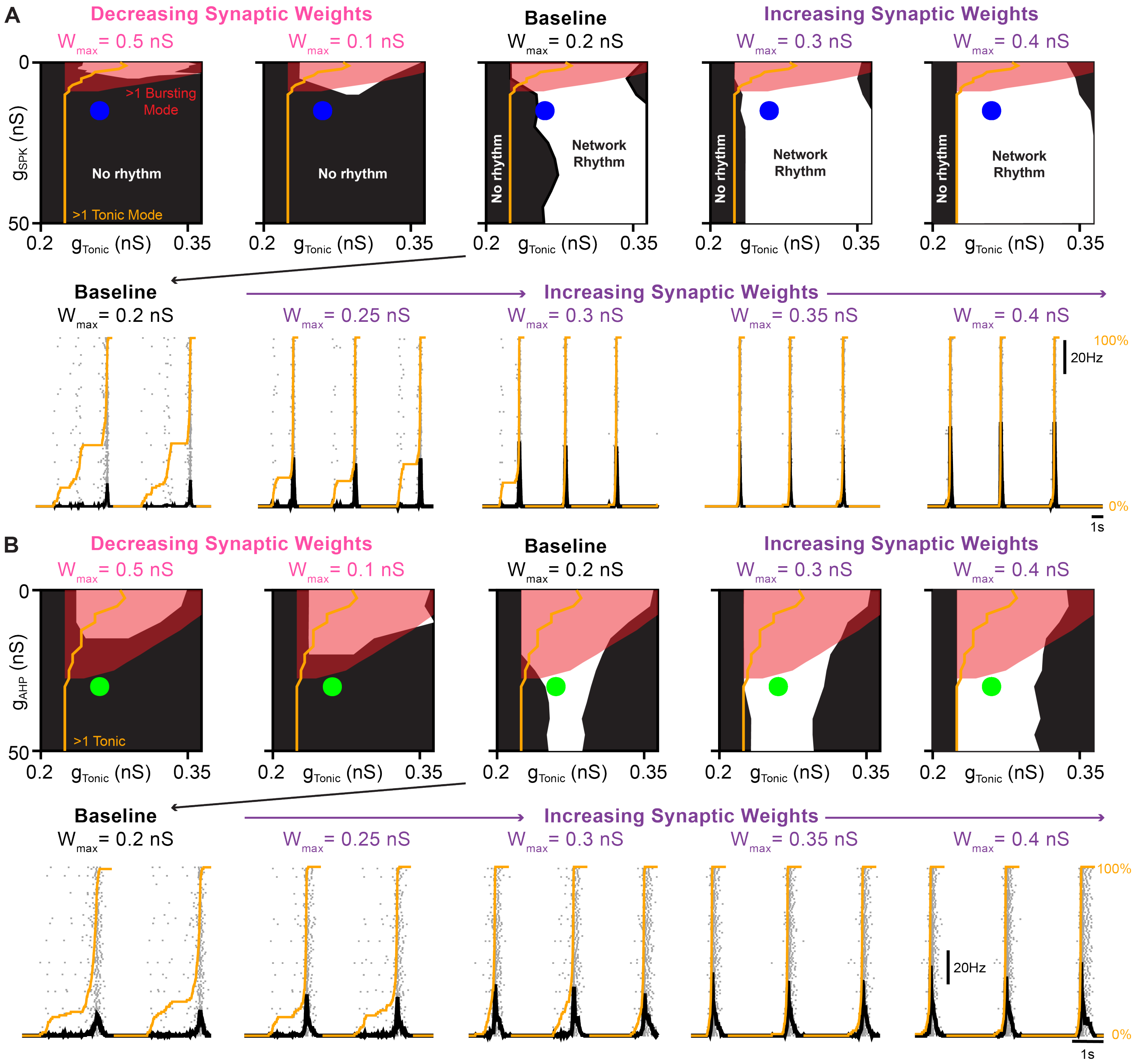

Figure 2-Figure Supplement 1. Interactions between spike shape, intrinsic bursting, and synaptic weight for network rhythmogenesis. In networks with (A) altered g_{SPK} or (B) altered g_{AHP} the parameter space supporting network rhythmogensis (white regions) was collapsed by decreasing synaptic weights and expanded by increasing synaptic weights. Blue ($g_{AHP} = 30nS$) and green ($g_{SPK} = 15nS$) dots correspond to g_{SPK}/g_{AHP} and g_{Tonic} values of representative traces at baseline and during increasing synaptic weight. Orange lines in example traces indicate the percentage of neurons in the network that have become active since the preceding network burst.

bioRxiv preprint doi: https://doi.org/10.1101/2023.10.30.564834; this version posted November 2, 2023. The copyright holder for this preprint (which was not certified by peer review) is the author/funder, who has granted bioRxiv a license to display the preprint in perpetuity. It is made available under aCC-BY 4.0 International license.

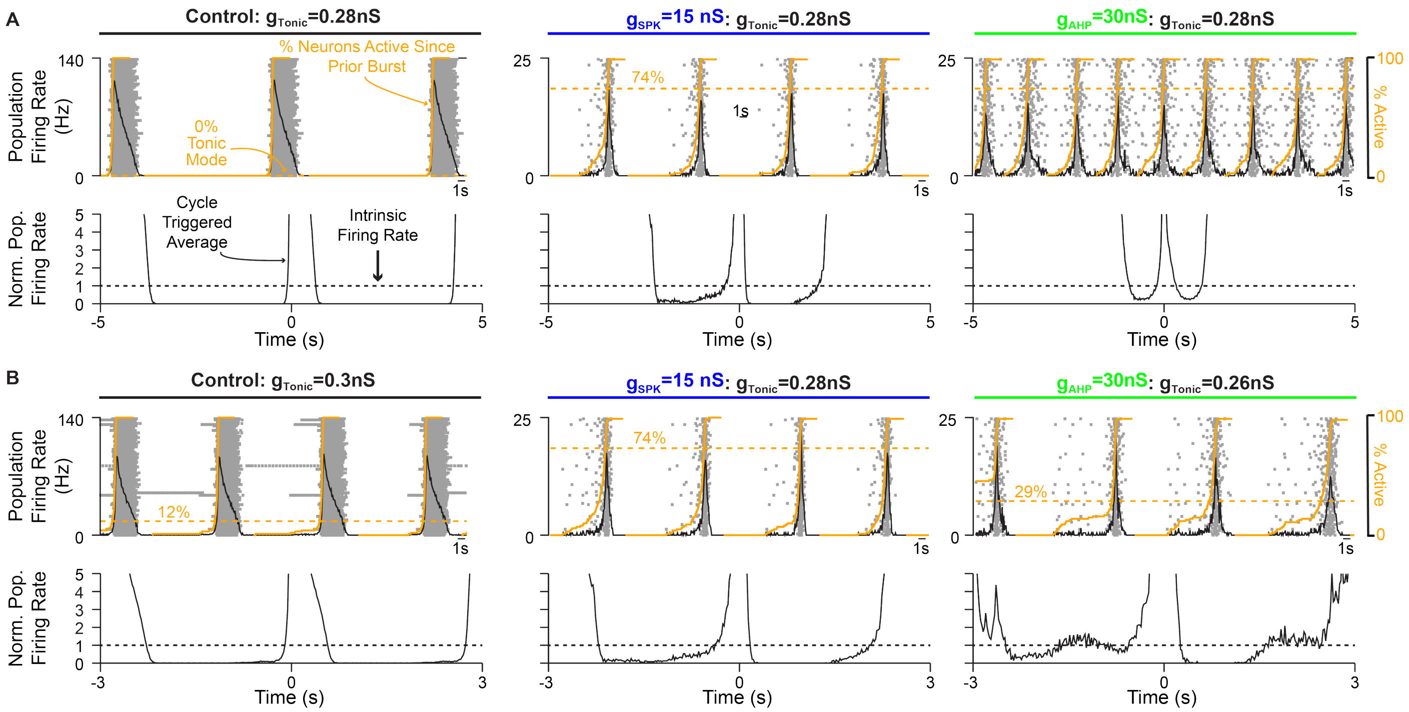

Figure 2-Figure Supplement 2. Relationship between pre-inspiratory spiking, the percentage of neurons in tonic spiking mode and the intrinsic network firing rate. Example traces (top) and cycle triggered averages (bottom) in networks with (A) fixed excitability (g_{Tonic}) or (B) altered excitability such that network frequencies are roughly equal ($\approx 3\,Hz$). Notice the emergence of pre-inspiratory spiking coincides with the transition of neurons into tonic mode due in the control network and in networks with altered spike shapes.

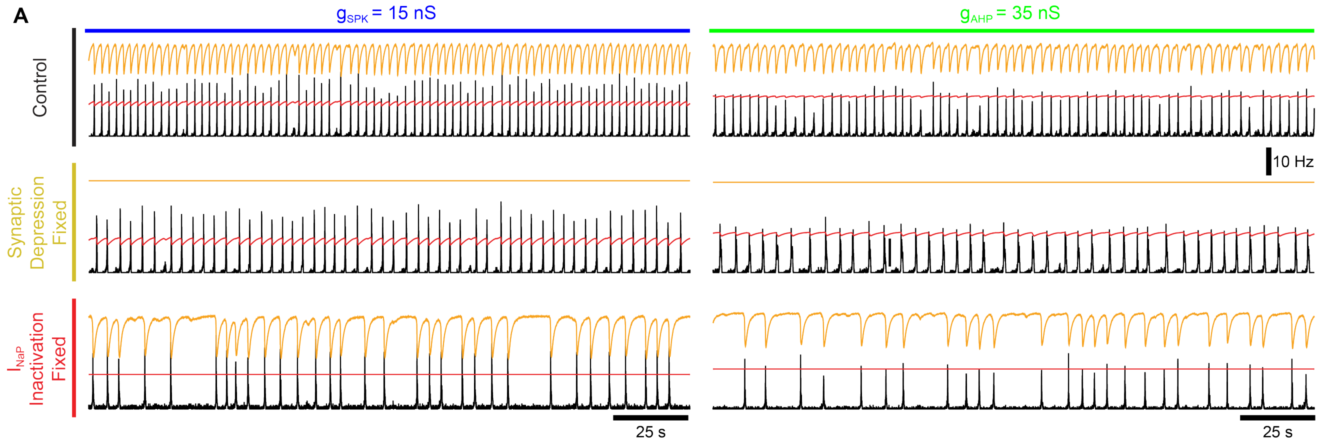

Figure 3 Supplement 1. Example network activity (firing rate) and corresponding synaptic depression (orange lines) and I_{NaP} inactivation (red lines) in networks with $g_{SPK} = 15\,nS$ (left) or $g_{AHP} = 35\,nS$ (right) under baseline conditions (top) or after fixing synaptic depression (middle) or I_{NaP} inactivation (bottom).

bioRxiv preprint doi: https://doi.org/10.1101/2023.10.30.564834; this version posted November 2, 2023. The copyright holder for this preprint (which was not certified by peer review) is the author/funder, who has granted bioRxiv a license to display the preprint in perpetuity. It is made available under aCC-BY 4.0 International license.

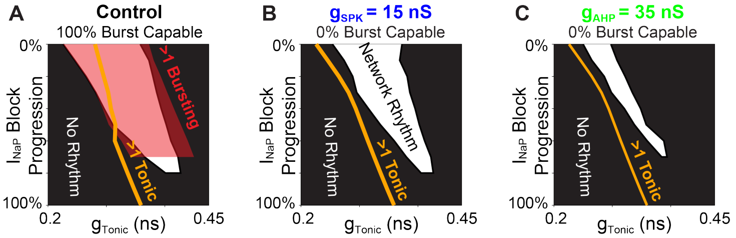

Figure 4 Supplement 1. Parameter space supporting intrinsic bursting (red) and network rhythmogenesis (white) as a function of excitability (g_{Tonic}) during progressive I_{NaP} block in (A) a control network with 100% of neurons initially burst capable ($g_{SPK} = g_{AHP} = 0$) and in networks with (B) $g_{SPK} = 15nS$ or (C) $g_{AHP} = 35nS$ to eliminate intrinsic bursting. Orange lines indicate g_{Tonic} value at which ≥ 1 neuron enters tonic spiking mode.

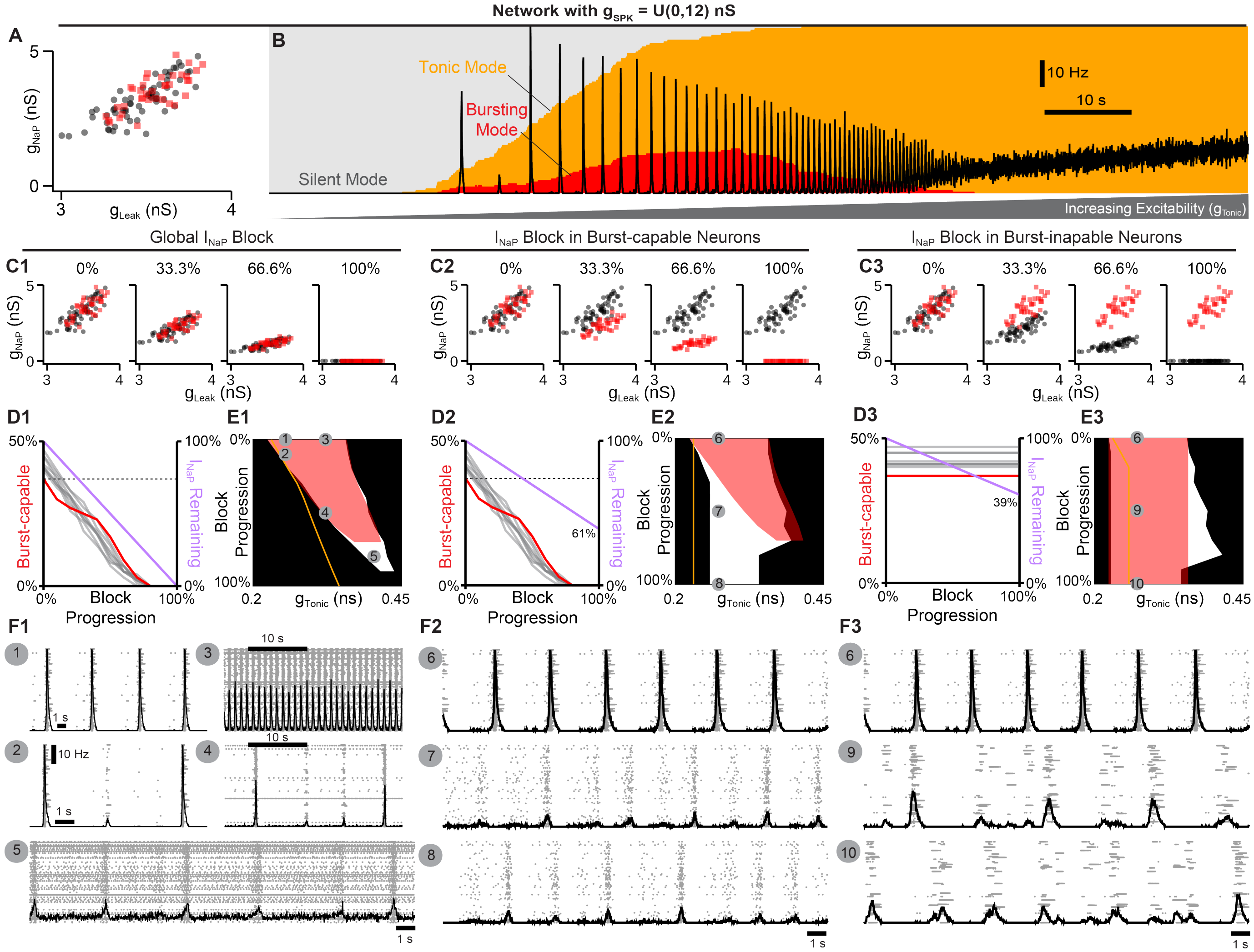

Figure 4 Supplement 2. Selective block of I_{NaP} in burst-capable or burst-incapable neurons has similar consequences for rhythm generation. (A) Distributions of g_{NaP} and g_{Leak} among burst-capable (red) and incapable (black) neurons in a network with $g_{SPK} = U(0,12)nS$. (B) Prevalence of silent, bursting, and tonic intrinsic cellular activities with overlaid network firing rate during increasing g_{Tonic} in the same network. (C1-3) Comparison of global I_{NaP} block (C1) vs. progressive I_{NaP} block specifically in neurons that are initially burst-capable (C2) or burst-incapable (C3). (D1-3) Fraction of the network that is burst-capable and amount of I_{NaP} remaining as a function of I_{NaP} block progression. (E1-3) Parameter space supporting intrinsic bursting (red) and network rhythmogenesis (white) as a function of excitability (g_{Tonic}) during progressive I_{NaP} block. (F1-F3) Raster plots and overlaid network firing rate corresponding to points 1-10 shown in E1-3.

bioRxiv preprint doi: https://doi.org/10.1101/2023.10.30.564834; this version posted November 2, 2023. The copyright holder for this preprint (which was not certified by peer review) is the author/funder, who has granted bioRxiv a license to display the preprint in perpetuity. It is made available under aCC-BY 4.0 International license.

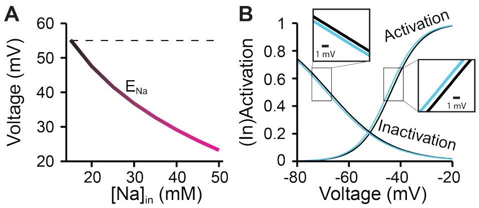

Figure 5 Supplement 1. Hypoxia related effects of (A) accumulating $[Na^+]_{in}$ on sodium reversal potential and (B) a hyperpolarizing shift in the (in)activation dynamics of spike generating sodium currents.

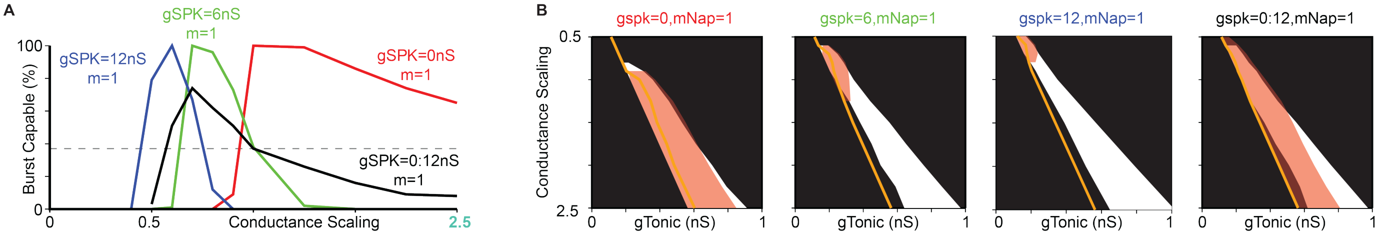

Figure 6 Supplement 1. Comparison of conductance scaling across networks with $g_{SPK} = 0\,nS, g_{SPK} = 6\,nS, g_{SPK} = 12\,nS$, or $g_{SPK} = U(0,12)\,nS$ showing (A) fraction of the network that is burst-capable, and (B) parameter spaces supporting intrinsic bursting (red) and network rhythmogenesis (white) as conductances are up- or down-scaled (Orange lines indicate g_{Tonic} where ≥ 1 neuron enters tonic spiking mode).

bioRxiv preprint doi: https://doi.org/10.1101/2023.10.30.564834; this version posted November 2, 2023. The copyright holder for this preprint (which was not certified by peer review) is the author/funder, who has granted bioRxiv a license to display the preprint in perpetuity. It is made available under aCC-BY 4.0 International license.

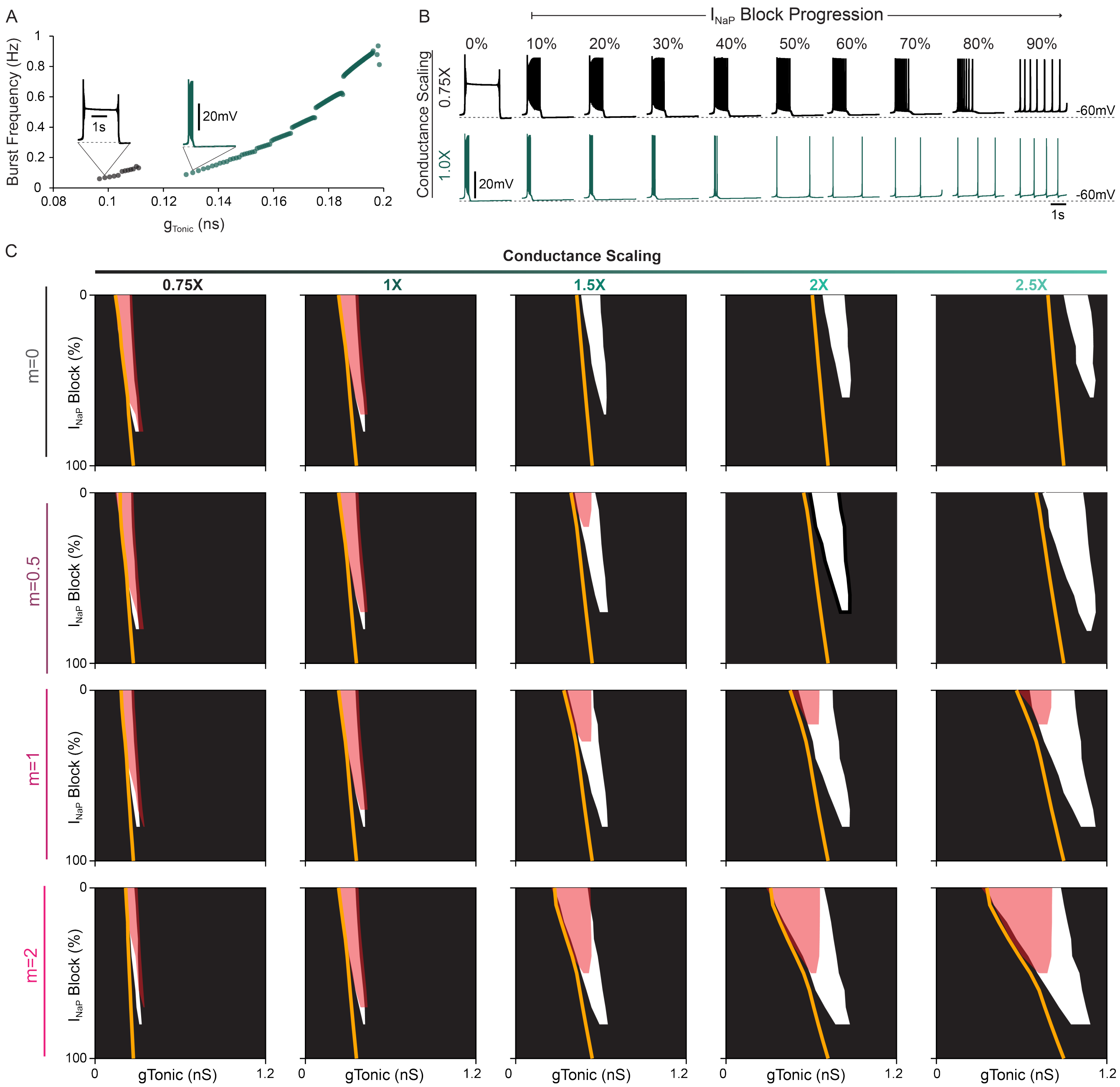

Figure 6 Supplement 2. (A) Relationship between excitability (g_{Tonic}) and burst frequency and (B) effect of simulated I_{NaP} block on intrinsic bursting capabilities for a neuron in with reduced conductance scaling (0.75X,m=1) compared to control scaling (1.0X,m=1). (C) Parameter space supporting network rhythmogenesis during progressive I_{NaP} block with scaled conductances.

bioRxiv preprint doi: https://doi.org/10.1101/2023.10.30.564834; this version posted November 2, 2023. The copyright holder for this preprint (which was not certified by peer review) is the author/funder, who has granted bioRxiv a license to display the preprint in perpetuity. It is made available under aCC-BY 4.0 International license.

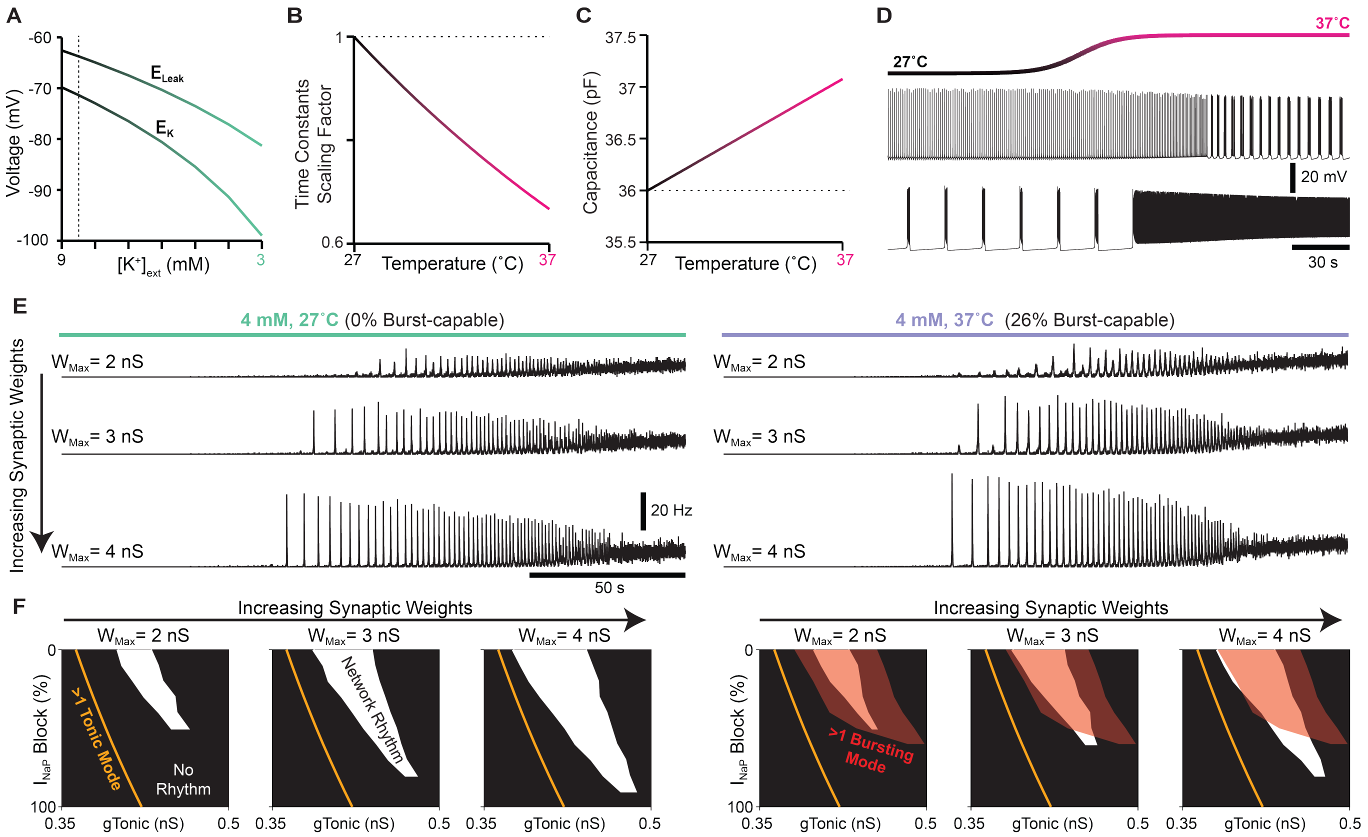

Figure 7 Supplement 1. Impact of extracellular potassium, temperature and synaptic weights on network properties and dynamics. (A) Relationship between the potassium (E_K) and leak (E_{Leak}) reversal potentials and extracellular potassium $[K^+]_{ext}$. Relationship between the scaling of time constants (B) and cellular capacitance (C) and the imposed temperature. (D) Example voltage traces illustrating the transition of a neuron from tonic to bursting mode and from bursting to tonic mode in response to an increase in temperature. (E) Effect of increases in synaptic weights on the network rhythm at physiological potassium and *in vitro* (left) or *in vivo* (right) temperatures. (F) Simulated I_{NaP} attenuation on network rhythms and intrinsic bursting.

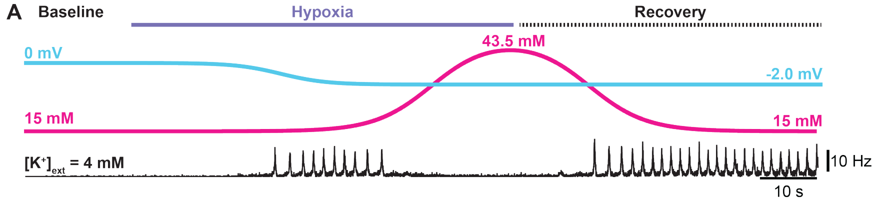

Figure 7 Supplement 2. Simulated hypoxia at physiological $[K^+]_{ext}$. (A) Network rhythm during transient hypoxia and recovery.
